# Atrial electromechanical coupling intervals in pregnant subjects

**DOI:** 10.5830/CVJA-2013-085

**Published:** 2014-02

**Authors:** Burak Altun, Gaz Emine, Ahmet Temi, Ahmet Barutcu, Yucel Colkesen, Ufuk Ozturk, Hakan Tasolar, Aysenur Cakir Gungor, Ahmet Uysal, Gurkan Acar, Murat Akkoyun

**Affiliations:** Department of Cardiology, Canakkale Onsekiz Mart University, Canakkale, Turkey; Department of Cardiology, Canakkale Onsekiz Mart University, Canakkale, Turkey; Department of Cardiology, Canakkale Onsekiz Mart University, Canakkale, Turkey; Department of Cardiology, Canakkale Onsekiz Mart University, Canakkale, Turkey; Department of Cardiology, Canakkale Onsekiz Mart University, Canakkale, Turkey; Department of Cardiology, Canakkale Onsekiz Mart University, Canakkale, Turkey; Department of Cardiology, Adiyaman University Training and Research Hospital, Adiyaman, Turkey; Department of Obstetrics and Gynecology, Canakkale Onsekiz Mart University, Canakkale, Turkey; Department of Obstetrics and Gynecology, Canakkale Onsekiz Mart University, Canakkale, Turkey; Department of Cardiology, Kahramanmaras Sutcu Imam University, Kahramanmaras, Turkey; Department of Cardiology, Kahramanmaras Sutcu Imam University, Kahramanmaras, Turkey

**Keywords:** atrial electromechanical coupling, pregnancy, tissue Doppler imaging

## Abstract

**Objective:**

The aim of this study was to evaluate atrial conduction abnormalities obtained by tissue Doppler imaging (TDI) and electrocardiogram analysis in pregnant subjects.

**Methods:**

A total of 30 pregnant subjects (28 ± 4 years) and 30 controls (28 ± 3 years) were included. Systolic and diastolic left ventricular (LV) function was measured using conventional echocardiography and TDI. Inter-atrial, intra-atrial and intra-left atrial electromechanical coupling (PA) intervals were measured with TDI. P-wave dispersion (PD) was calculated from a 12-lead electrocardiogram.

**Results:**

Atrial electromechanical coupling at the septal and left lateral mitral annulus (PA septal, PA lateral) was significantly prolonged in pregnant subjects (62.1 ± 2.7 vs 55.3 ± 3.2 ms, *p* < 0.001; 45.7 ± 2.5 vs 43.1 ± 2.7 ms, *p* < 0.001, respectively). Inter-atrial (PA lateral – PA tricuspid), intra-atrial (PA septum – PA tricuspid) and intra-left atrial (PA lateral – PA septum) electromechanical coupling intervals, maximum P-wave (P_max_) duration and PD were significantly longer in the pregnant subjects (26.4 ± 4.0 vs 20.2 ± 3.6 ms, *p* < 0.001; 10.0 ± 2.0 vs 8.0 ± 2.6 ms, *p* = 0.002; 16.4 ± 3.3 vs 12.2 ± 3.0 ms, *p* < 0.001; 103.1 ± 5.4 vs 96.8 ± 7.4 ms, *p* < 0.001; 50.7 ± 6.8 vs 41.6 ± 5.5 ms, *p* < 0.001, respectively). We found a significant positive correlation between inter-atrial and intra-left atrial electromechanical coupling intervals and P_max_ (*r* = 0.282, *p* = 0.029, *r* = 0.378, *p* = 0.003, respectively).

**Conclusion:**

This study showed that atrial electromechanical coupling intervals and PD, which are predictors of AF, were longer in pregnant subjects and this may cause an increased risk of AF in pregnancy.

## Abstract

Atrial fibrillation (AF), which is the most common cardiac arrhythmia, may cause serious symptoms and impair quality of life.^1^ The development of AF is associated with many risk factors, including age, male gender, hypertension, heart failure, valvular disease, diabetes mellitus (DM) and left atrial (LA) enlargement.^2^-^4^ Electrical and/or mechanical remodelling of the atria is thought to be a pathophysiological characteristic of AF.^5^

The pregnant state may be pro-dysrhythmic. This is related to the cardiovascular, hormonal, haemodynamic and autonomic changes during healthy pregnancy. Levels of oestrogen and β-human chorionic gonadotropin increase dramatically. Haemodynamic changes include an increase in circulating blood volume, which increases cardiac output. This results in myocardial stretch and an increase in cardiac end-diastolic volume. High plasma catecholamine concentrations and adrenergic receptor sensitivity increase sympathetic tone. All these changes in pregnant women may make them more prone to dysrhythmogenesis.^6^

Most pregnant women complain of palpitations, dizziness and even syncope, but these symptoms are rarely associated with cardiac dysrhythmias. AF is the most common clinically significant cardiac arrhythmia in the general population but it is rarely seen in pregnant women. When it occurs, it can represent a benign, self-limited lone episode of AF or may be secondary to congenital or rheumatic valvular disease, hypertrophic cardiomyopathy, thyroid disease, or pre-excitation syndrome.

Two simple electrocardiogram (ECG) markers, namely maximum P-wave duration (Pmax) and P-wave dispersion (PD), have been used to evaluate intra- and inter-atrial conduction times and the inhomogeneous propagation of sinus impulses, which are well-known electrophysiological characteristics of the atrium prone to fibrillation.^7^,^8^ Prolonged Pmax and PD have been reported to represent an increased risk for AF in patients with no underlying heart disease.^7^,^8^ Besides, evidence from laboratory and epidemiological research suggests that systemic inflammation may play a role in AF aetiology.^9^ It has also been demonstrated that atrial electromechanical coupling, measured by tissue Doppler imaging (TDI), as significantly longer in patients with paroxysmal AF than in control groups.^10^,^11^

To our knowledge, no study evaluating PD and atrial electromechanical coupling has been investigated in pregnant subjects without additional systemic disease. Therefore, in this study we aimed to examine atrial electromechanical coupling and PD, reflecting inter-atrial conduction times in pregnant subjects.

## Methods

We consecutively studied 40 pregnant subjects. Eight were excluded from the study, because of thyroid dysfunction in three subjects, DM in three, unclearly identifiable P waves in two, and bundle branch block in two. The study population was composed of 30 pregnant subjects (mean age 28 ± 4 years) and 30 age-matched controls (mean age 28 ± 3 years). All the pregnant women were in the second trimester between 18 and 23 weeks. Physical examination, medical history of the patients and blood biochemistry were evaluated in both groups to exclude systemic diseases.

Subjects with coronary artery disease, heart failure, rheumatic valve disease, primary cardiomyopathy, DM, hypertension, thyroid dysfunction, any previous arrhythmia, anaemia, electrolyte imbalance, chronic lung disease, and bundle branch block and atrio-ventricular conduction abnormalities on ECG were excluded from the study. Also, ECGs without clearly identifiable P waves were excluded from the PD analysis using standard 12-lead surface ECGs.

All of the patients were in sinus rhythm and none was taking medications such as anti-arrhythmics, tricyclic antidepressants, antihistamines and antipsychotics. All patients signed informed consent form. The local ethics committee approved the study.

Two-dimensional, M-mode, pulsed and colour-flow Doppler echocardiographic examinations of all subjects were performed by the same examiner with a commercially available machine (Vivid 7 pro, GE, Horten, Norway, 2–4 mHz phased array transducer). During the echocardiography, a one-lead electrocardiogram was recorded continuously.

M-mode measurements were performed according to the criteria of the American Society of Echocardiography.^12^ LA diameter, and LV end-systolic and end-diastolic diameters were measured. LV ejection fraction (EF) was estimated using Simpson’s rule. LV mass was calculated with the Devereux formula.^13^

Conventional Doppler echocardiography was performed and pulsed-wave mitral flow velocities were measured from the apical four-chamber view by inserting a sample volume to the mitral leaflet tips. Mitral early diastolic velocity (E, cm/s), late diastolic velocity (A, cm/s), E/A ratio (E/A), E deceleration time (DT, ms), and isovolumetric relaxation time (IVRT, ms) were determined. Each representative value was obtained from the average of three measurements. The operator was blinded to the clinical details and results of the other investigations of each pregnant subject and control.

Tissue Doppler imaging echocardiography was performed with transducer frequencies of 3.5–4.0 MHz, adjusting the spectral pulsed Doppler signal filters until a Nyquist limit of 15–20 cm/s was reached and using the minimal optimal gain. The monitor sweep speed was set at 50–100 mm/s to optimise the spectral display of myocardial velocities.

Myocardial peak systolic (Sm, cm/s), and early (Em, cm/s) and late (Am, cm/s) diastolic velocities, Em/Am ratio, isovolumetric contraction time (ICT, ms), isovolumetric relaxation time (IRT, ms) and ejection time (ET, ms) were obtained by placing a tissue Doppler sample volume in the basal segments of the anterior, inferior, lateral, and septal wall.^14^ The tricuspid annular motion was recorded at the right ventricular (RV) free wall. Myocardial performance index (MPI) was calculated using the (ICT + IRT)/ET formula.^15^ By calculating the arithmetical mean value of the segmentary values, mean LV Sm, Em, mean Am, mean MPI, and Em/Am values were obtained.

Tissue Doppler velocities therefore represent an average of the basal segments of the anterior, inferior, lateral and septal walls. Also, the E/Em ratio, an important non-invasive marker of pulmonary capillary wedge pressure and LV filling pressure, was calculated. Diastolic dysfunction was defined according to the guidelines of the European Association of Echocardiography/American Society of Echocardiography as the presence of septal Em < 8 cm/s, lateral Em < 10 cm/s and LA volume ≥ 34 ml/m^2^.^16^

**Fig. 1.**
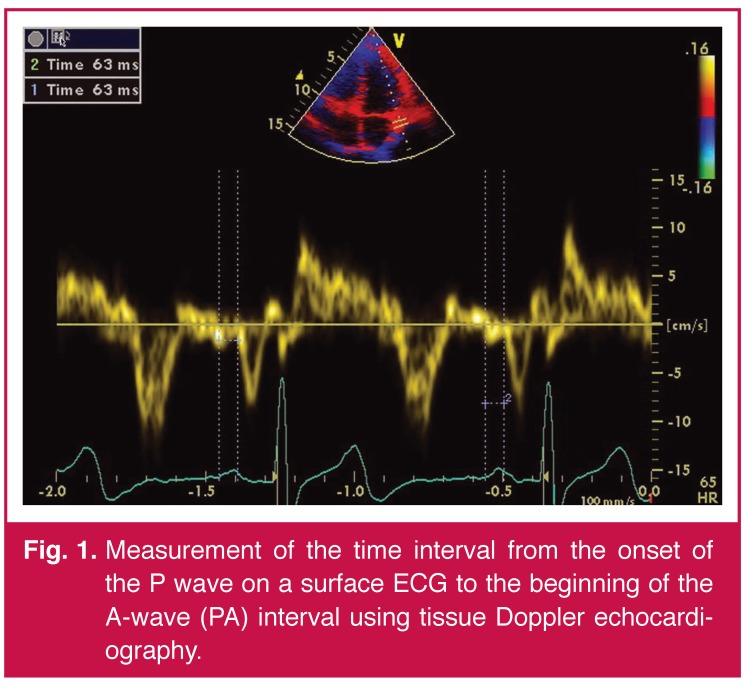
Measurement of the time interval from the onset of the P wave on a surface ECG to the beginning of the A-wave (PA) interval using tissue Doppler echocardiography.

Atrial electromechanical coupling was determined as follows. In an apical four-chamber view, the pulsed Doppler sample volume was placed at the level of the LV lateral mitral annulus, septal mitral annulus, and RV tricuspid annulus. The time interval from the onset of the P wave on a surface ECG to the beginning of the late diastolic wave (Am), which is termed PA, was obtained from the lateral mitral annulus (PA lateral), septal mitral annulus (PA septal), and RV tricuspid annulus (PA tricuspid) (Fig. 1). The difference between PA lateral and PA tricuspid (PA lateral – PA tricuspid) was defined as the interatrial electromechanical coupling interval; PA septum and PA tricuspid (PA septum – PA tricuspid) was defined as intra-atrial electromechanical coupling interval; and the difference between PA septal and PA lateral (PA septal – PA lateral ) was defined as intra-left atrial electromechanical coupling interval.^18^

P-wave dispersion was measured on 12-lead ECGs. All standard 12-lead ECGs were obtained simultaneously using a recorder (Hewlett Packard, Pagewriter) set at a 50-mm/s paper speed and 1-mV/cm standardisation. ECG measurements were evaluated on the same day, in a one-month period in our routine practice. A single cardiologist, who was blinded to the clinical status of the subjects, measured ECG intervals. To decrease the error measurements, P-wave analysis was done with calipers and magnifying glass.

The beginning of the P wave was defined as the point where the initial deflection of the P wave crossed the isoelectric line, and the end of the P wave was defined as the point where the final deflection of the P wave crossed the isoelectric line. ECGs with measurable P waves in less than 10 leads were also excluded from the analysis. In all patients, derivations were excluded if the beginning or ending of the P wave could not be clearly identified. PD was calculated by subtracting the minimum P-wave duration (P_min_) from the P_max_.

## Statistical analysis

Statistical analyses were performed using SPPS software (version 15.0, SPSS, Chicago, Illinois, USA). An assessment of the normality was done initially with Kolmogorov–Smirnov. All numerical data were expressed as mean ± standard deviation or median (interquartile range). Groups were compared by Mann–Whitney *U*- or student *t*-tests. The relationship between parameters was calculated using Pearson correlation analysis. The intra-observer variability for the detection of atrial electromechanical coupling was evaluated by Spearman’s correlation; *p*-values < 0.05 were considered significant.

## Results

The laboratory and clinical characteristics of the subjects are presented in Table 1. Mean age, heart rate, systolic and diastolic BP, and glucose levels were similar in both groups. However, haemoglobin levels were significantly lower in the pregnant subjects (12.6 ± 1.3 vs 13.3 ± 1.1 mg/dl, *p* = 0.046) and body mass index was higher in the pregnant subjects (26.8 ± 2.4 vs 24.4 ± 3.8 kg/m^2^, *p* = 0.006)

**Table 1:** Clinical and laboratory characteristics of the subjects

	*Pregnant subjects*	*Controls*	p*-value*
Age (years)	28 ± 4	28 ± 3	0.817
Heart rate (bpm)	83.0 ± 10.8	80.2 ± 9.5	0.293
BMI (kg/m^2^)	26.8 ± 2.4	24.4 ± 3.8	0.006
Glucose (mg/dl)	89.9 ± 10.0	90.9 ± 10.9	0.706
Hgb (mg/dl)	12.6 ± 1.3	13.3 ± 1.1	0.046
SBP (mmHg)	116.5 ± 12.3	116.5 ± 10.5	0.991
DBP (mmHg)	74.6 ± 7.1	74.2 ± 7.0	0.828

BMI: body mass index, Hgb: haemoglobin, SBP: systolic blood pressure, DBP: diastolic blood pressure.

Echocardiographic results are listed in Table 2. The LV end-diastolic and end-systolic dimensions, inter-ventricular septum thickness, LV posterior wall thickness, LV ejection fraction, LA diameter, and E velocity were similar in both groups. However, the A velocity and DT were significantly higher (81.3 ± 17.5 vs 65.0 ± 12.5 ms, *p* < 0.0001; 204.8 ± 20.8 vs 193.5 ± 15.9 ms, *p* = 0.007, respectively) in the pregnant subjects than the controls. There were no significant differences between the two groups with regard to Sm, Em, Am, IRT, ICT, ET and MPI values (Table 2).

**Table 2:** Echocardiographic and tissue Doppler echocardiographic parameters

*Parameters*	*Pregnant subjects*	*Controls*	p*-value*
Echocardiographic parameters			
LVEDD (mm)	45.4 ± 3.2	45.9 ± 4.0	0.579
LVESD (mm)	27.7 ± 3.1	28.9 ± 3.2	0.170
IVS thickness (mm)	9.8 ± 0.6	9.6 ± 1.1	0.427
PW thickness (mm)	8.6 ± 0.6	8.6 ± 1.2	0.900
LV mass	181.8 ± 43.8	160.5 ± 36.9	0.047
LV EF (%)	68.8 ± 6.4	66.7 ± 6.3	0.208
Left atrium dimension (mm)	33.0 ± 4.0	32.6 ± 4.9	0.753
Mitral E velocity (cm/s)	83.4 ± 18.3	79.9 ± 14.8	0.416
Mitral A velocity (cm/s)	81.3 ± 17.5	65.0 ± 12.5	< 0.001
DT (ms)	204.8 ± 20.8	193.5 ± 15.9	0.007
IVRT (ms)	90.1 ± 12.3	86.2 ± 5.4	0.223
Tissue Doppler parameters			
Sm (cm/s)	11.1 ± 2.4	10.6 ± 1.7	0.395
Em (cm/s)	12.8 ± 4.0	13.6 ± 3.3	0.371
Am (cm/s)	12.1 ± 2.4	10.8 ± 2.7	0.070
E/Em	6.9 ± 1.9	6.0 ± 1.3	0.046
ICT (ms)	69.8 ± 19.2	72.5 ± 14.7	0.545
IRT (ms)	64.1 ± 10.2	67.0 ± 11.6	0.321
ET (ms)	267.1 ± 31.2	281.8 ± 29.2	0.065
MPI	50.4 ± 9.2	49.8 ± 7.8	0.785

LV: left ventricular; LVEDD: LV end-diastolic dimension; LVESD: LV end-systolic dimension; IVS: interventricular septum; PW: posterior wall; EF: ejection fraction; DT: mitral E-wave deceleration time; IRT: isovolumetric relaxation time; Sm: mean LV systolic myocardial velocity; Em: mean LV myocardial early diastolic velocity; Am: mean LV myocardial late diastolic velocity; ICT: mean LV isovolumetric contraction time; IRT: mean LV isovolumetric relaxation time; ET: mean LV ejection time; MPI: myocardial performance index.

Intra-observer variability was assessed in 20 selected subjects at random from the patient study group by repeating the measurements under the same baseline conditions. Intra-observer coefficients of variation for echocardiographic measurements were found to be < 5% and non-significant.

The atrial electromechanical coupling parameters of different sites measured by TDI and P-wave measurements are shown in Table 3. The PA lateral and PA septum were significantly higher in the pregnant subjects compared with the controls (62.1 ± 2.7 vs 55.3 ± 3.2 ms, 45.7 ± 2.5 vs 43.1 ± 2.7 ms, *p* < 0.001). The PA tricuspid did not differ significantly between the groups (*p* > 0.05). Furthermore, inter-atrial, intra-atrial and intra-left atrial electromechanical coupling intervals were also prolonged in the pregnant subjects compared the controls (26.4 ± 4.0 vs 20.2 ± 3.6 ms, *p* < 0.001; 10.0 ± 2.0 vs 8.0 ± 2.6 ms, *p* = 0.002; 16.4 ± 3.3 vs 12.2 ± 3.0 ms, *p* < 0.001, respectively).

**Table 3:** Comparison of the electrocardiographic and electromechanical coupling parameters

	*Pregnant subjects*	*Controls*	p*-value*
Maximum P-wave duration (ms)	103.1 ± 5.4	96.8 ± 7.4	< 0.001
Minimum P-wave duration (ms)	52.4 ± 6.3	55.1 ± 5.7	0.090
P-wave dispersion (ms)	50.7 ± 6.8	41.6 ± 5.5	< 0.001
PA lateral (ms)	62.1 ± 2.7	55.3 ± 3.2	< 0.001
PA septal (ms)	45.7 ± 2.5	43.1 ± 2.7	< 0.001
PA tricuspid (ms)	35.7 ± 2.7	35.1 ± 3.2	0.440
PA lateral – PA tricuspid*	26.4 ± 4.0	20.2 ± 3.6	< 0.001
PA septal – PA tricuspid**	10.0 ± 2.0	8.0 ± 2.6	0.002
PA lateral – PA septal***	16.4 ± 3.3	12.2 ± 3.0	< 0.00

PA: time interval from the onset of the P wave on the surface ECG to the beginning of the A-wave interval with tissue Doppler imaging. *Inter-atrial electromechanical coupling interval, **Intra-atrial electromechanical coupling interval, ***Intra-left atrial electromechanical coupling interval.

P-wave measurements are given in Table 3. Both the P_max_ and the PD were significantly longer in the pregnant subjects (103.1 ± 5.4 vs 96.8 ± 7.4 ms, *p* < 0.001; 50.7 ± 6.8 vs 41.6 ± 5.5 ms, *p* < 0.001, respectively). In addition, a significant positive correlation was found between inter-atrial and intra-left atrial electromechanical coupling interval and P_max_ (*r* = 0.282, *p* = 0.029, *r* = 0.378, *p* = 0.003, respectively).

**Fig. 2.**
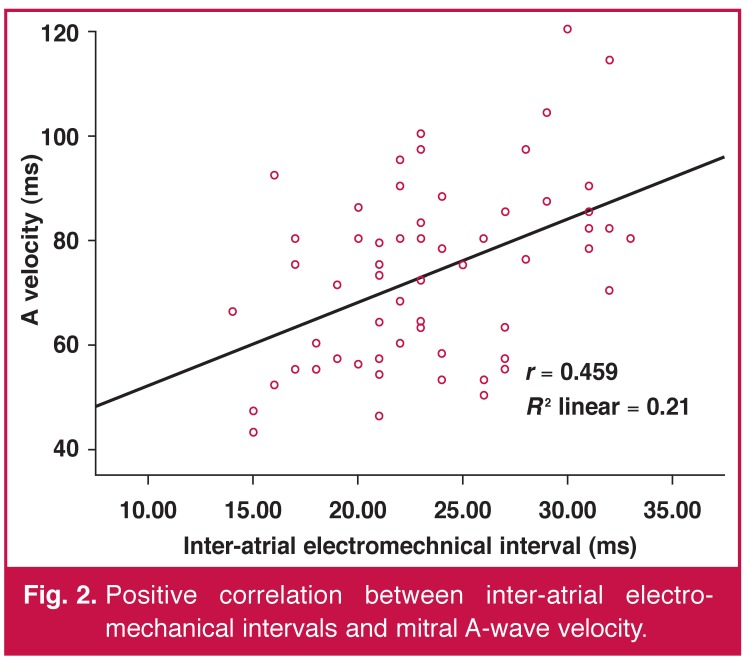
Positive correlation between inter-atrial electromechanical intervals and mitral A-wave velocity.

In correlation analysis, no relationship was detected between the atrial electromechanical coupling parameters and clinical data such as age, heart rate, systolic and diastolic BP. However, there were significant correlations between the inter-atrial and intra-left atrial electromechanical coupling interval and the A velocity (*r* = 0.459, *p* < 0.001, *r* = 0.448, *p* < 0.001, respectively) (Fig. 2).

## Discussion

There were three major findings of this study. The intra- and inter-atrial, and intra-left atrial electromechanical coupling intervals were significantly higher in the pregnant subjects. The P_max_ and PD were also significantly higher in the pregnant subjects and there was a significant correlation between the inter-atrial and the intra-left atrial electromechanical coupling interval P_max_.

Clapp *et al.* reported a progressive increase in all cardiac chamber dimensions in pregnancy.,^19^ However, Katz *et al.* found no statistically significant differences between left atrial diameter and left ventricular internal diastolic diameter (LVIDD) in pregnant subjects in the second trimester and at 12 weeks postpartum.,^20^ In our study, there were no differences between the LA diameter and LVIDD in pregnant subjects compared to the controls, which is in line with the previous study.

Increases in maternal blood volume, cardiac output and heart rate are seen during pregnancy. These mechanisms affect the refractory period and conduction velocity. Schwartz and Priori found that stress and anxiety also caused arrhythmogenic effects by acting on the sympathetic nervous system.,^21^

Hormonal changes also seem to play an important role in arrhythmias during pregnancy. Gleicher *et al.* found that oestrogens increased the excitability and frequency of action potentials in uterine muscle tissue during pregnancy.,^22^ This increased adrenergic sensitivity may play a role in the genesis of arrhythmias by modifying the refractory period and conduction velocity in the re-entrant circuit.

When left ventricular diastolic dysfunction occurs, emptying of the left atrium is also impaired. Following impaired left ventricular diastolic relaxation, there is increased atrial contribution to the mitral flow in the left ventricular diastolic flow, thus leading to myocardial overstretching and enlargement.,^23^ In our study, DT and A velocity were significantly higher in the pregnant subjects, but IVRT, IRT and ET were similar to the controls. The left atrium diameter is known to be correlated with cardiovascular events and a risk factor for AF.,^24^ These volumetric changes also constitute a high risk for AF.

There are conflicting results on this topic. In our study, the diameters of the pregnant subjects were similar to those of the controls, in line with our previous studies,^24^-^26^ whereas some studies found increased LA dimension in patients with diastolic dysfunction and increased coupling parameters.^27^,^28^ We believe there is a need for large-scale studies to shed light on this discrepancy.

PD is related to non-homogenous and interrupted conduction of sinus impulses intra- and inter-atrially. Currently, prolonged P_max_, increased PD and atrial conduction disorders are associated with a higher risk of paroxysmal atrial tachyarrhythmias.^7^,^8^ Therefore, it has been suggested that PD can be used in the diagnosis of patients with a high risk of AF.^7^,^8^ It was moreover shown that PD was prolonged in chronic, inflammatory and rheumatic diseases, such as rheumatoid arthritis and Behcet’s disease.^29^,^30^

In another study, PD was increased in pregnancy due to shortening of the minimum P-wave length and it reached its longest length in the third trimester. Pregnancy also had no effect on P_max_.^31^ In our study, P_max_ and PD were longer in pregnant subjects than in the controls, and this increased PD was related to prolonged P_max_.

There are several ways to measure total atrial conduction time; one is signal-averaged ECG, which is the gold-standard technique, but it requires special hardware and is a longer technique. For this reason, measurement of total atrial conduction time by signal-averaged ECG is not often used in clinical practice.

Mercadier *et al.* have shown PA TDI to be an easy, fast and reliable method to measure total atrial electrical activation time.,^23^ PA TDI duration is a readily available echocardiographic tool to estimate total atrial conduction time and it can easily be measured by all cardiologists. This novel echocardiographic tool has been validated by the P-wave duration on signal-averaged electrocardiography.^32^

Atrial conduction time can be measured by both invasive and non-invasive methods.^33^ Prolongation of atrial conduction time, as measured by TDI, is an independent predictor of new-onset or recurrent AF.^34^,^3^ Several studies have found that atrial conduction time measured by TDI increases in patients with various diseases, such as type 1DM,^25^ dilated cardiomyopathy,^36^ and ankylosing spondilitis.^26^

Hormonal action may play a role in atrial conduction and depolarisation. Increased oestradiol levels in pregrant women may contribute to a longer PD. There are no data comparing PD in men and women.

In our study, we demonstrated that atrial electromechanical coupling intervals, Pmax and PD, which is a non-invasive technique providing estimated risk of AF in sinus rhythm, were significantly more prolonged in pregnant subjects than in the controls. Our study is the first of its kind to investigate the relationship of atrial electromechanical coupling with pregnancy. We consider that prolonged PD may be related to volume overload secondary to pregnancy. We also believe that larger studies are needed to clarify this issue.

This study had several limitations. It was a cross-sectional study and the population size was relatively small. Intra-observer variability of measurement of PA interval was not investigated. Patients could not be followed up prospectively in terms of detection of long-term cardiac arrhythmia. Other limitations were the use of manual ECG measurements, absence of Holter monitoring, and lack of electrophysiological evaluation. The absence of strain rate parameters was another potential limitation of our study.

## Conclusion

This study demonstrated that atrial electromechanical coupling intervals and PD, which are predictors of AF, were prolonged in the pregnant subjects. These results suggest that longer atrial electromechanical coupling intervals may cause an increased risk of AF in pregnancy.
